# Synthesis of ^68^Ga-Labeled cNGR-Based Glycopeptides and In Vivo Evaluation by PET Imaging

**DOI:** 10.3390/pharmaceutics13122103

**Published:** 2021-12-07

**Authors:** Barbara Gyuricza, Judit P. Szabó, Viktória Arató, Noémi Dénes, Ágnes Szűcs, Katalin Berta, Adrienn Kis, Dániel Szücs, Viktória Forgács, Dezső Szikra, István Kertész, György Trencsényi, Anikó Fekete

**Affiliations:** 1 Division of Nuclear Medicine and Translational Imaging, Department of Medical Imaging, Faculty of Medicine, University of Debrecen, Nagyerdei krt. 98., H-4032 Debrecen, Hungary; gyuricza.barbara@med.unideb.hu (B.G.); szabo.judit@med.unideb.hu (J.P.S.); arato.viktoria@med.unideb.hu (V.A.); denes.noemi@med.unideb.hu (N.D.); szucsagnes97@gmail.com (Á.S.); berta.katalin@med.unideb.hu (K.B.); kis.adrienn@med.unideb.hu (A.K.); szucs.daniel@science.unideb.hu (D.S.); forgacs.viktoria@med.unideb.hu (V.F.); szikra.dezso@med.unideb.hu (D.S.); kertesz.istvan@med.unideb.hu (I.K.); trencsenyi.gyorgy@med.unideb.hu (G.T.); 2 Doctoral School of Chemistry, Faculty of Science and Technology, University of Debrecen, Egyetem tér 1., H-4032 Debrecen, Hungary; 3 Doctoral School of Clinical Medicine, Faculty of Medicine, University of Debrecen, Nagyerdei krt. 98., H-4032 Debrecen, Hungary; 4 Doctoral School of Pharmaceutical Sciences, Faculty of Pharmacy, University of Debrecen, Nagyerdei krt. 98., H-4032 Debrecen, Hungary; 5 Doctoral School of Molecular Medicine, Faculty of Medicine, University of Debrecen, Egyetem tér 1., H-4032 Debrecen, Hungary

**Keywords:** radiopharmaceuticals, gallium-68, positron emission tomography (PET), aminopeptidase N receptor (APN/CD13), NGR peptide

## Abstract

Tumor hypoxia induces angiogenesis, which is required for tumor cell survival. The aminopeptidase N receptor (APN/CD13) is an excellent marker of angiogenesis since it is overexpressed in angiogenic blood vessels and in tumor cells. Asparagine-glycine-arginine (NGR) peptide analogs bind selectively to the APN/CD13 recepto, therefore, they are important vector molecules in the development of a PET radiotracer which is capable of detecting APN-rich tumors. To investigate the effect of glycosylation and pegylation on in-vivo efficacy of an NGR-based radiotracer, two ^68^Ga-labeled radioglycopeptides were synthesized. A lactosamine derivative was applied to glycosylation of the NGR derivative and PEG_4_ moiety was used for pegylation. The receptor targeting potential and biodistribution of the radiopeptides were evaluated with in vivo PET imaging studies and ex vivo tissue distribution studies using B16-F10 melanoma tumor-bearing mice. According to these studies, all synthesized radiopeptides were capable of detecting APN expression in B16-F10 melanoma tumor. In addition, lower hepatic uptake, higher tumor-to background (T/M) ratio and prolonged circulation time were observed for the novel [^68^Ga]-**10** radiotracer due to pegylation and glycosylation, resulting in more contrasting PET imaging. These in vivo PET imaging results correlated well with the ex vivo tissue distribution data.

## 1. Introduction

Initially, small tumors do not have their own vascular network; therefore, tumor growth can stop due to a lack of oxygen and nutrients. However, oxygen deficiency induces angiogenesis and new capillaries are formed from existing vessels to allow tumor cells to survive [1]. Since angiogenesis leads to tumor growth and malignant development, inhibiting of tumor neovascularization is a valuable approach to anticancer therapy. This method requires biomarkers that are expressed in activated and metastatic vessels but are not found or are present in very small amounts in normal vessels.

Previous studies have revealed that among these molecules, aminopeptidase N receptor (APN, also known as CD13) is a suitable target for the detection and treatment of various cancer cells [2]. APN/CD13 is a Zn^2+^-dependent membrane-bound enzyme that degrades proteins and peptides with an *N*-terminal amino acid and is a regulator of malignancies, such as tumor cell invasion, differentiation, proliferation, apoptosis, and angiogenesis. The APN/CD13 is highly expressed in angiogenic blood vessels and in neoplastic cells and its expression level correlates with cancer progression. Therefore, determining the level of this receptor is suitable for diagnosing APN/CD13-positive tumors and monitoring the response to anti-cancer therapy. Numerous APN/CD13-positive solid tumor types are well-known, such as melanoma, lung, prostate, and ovarian cancer [2]. Molecules containing asparagine-glycine-arginine tripeptide sequence (NGR) bind to APN/CD13 receptor with high selectivity established by Pasqualini and et al. [3]. Accordingly, NGR analogs are promising vector molecules for delivering radioactive isotopes as well as chemotherapeutic drugs to APN/CD13 positive tumor cells due to their high binding affinity [4,5,6,7]. Some NGR conjugated chemotherapeutic agents have been investigated in clinical trials [8,9,10].

Positron emission tomography (PET) is a non-invasive functional imaging technique which enables the early detection of the pathological processes in the human body using in vivo, real-time measurements. The positron emitting ^68^Ga isotope possesses suitable nuclear properties (89% β^+^; t_1/2_ = 67.7 min; E_av_(β^+^) = 740 keV) for PET imaging, and the radiolabeling reaction of this radiometal can be performed as a practical one-pot synthesis using a chelator-containing precursor with high radiochemical yield and purity. Efficient use of ^68^Ga-labeled peptides in neuroendocrine tumor diagnostics stimulated the further development and clinical application of ^68^Ga-labeled diagnostic tracers for PET (e.g., ^68^Ga-DOTA-TOC and ^68^Ga-DOTA-TATE) [11,12]. To date, the synthesis and in vivo evaluation of some radiometal-labeled NGR peptides by PET imaging have been reported [13,14,15,16].

Tumor-targeting, peptide-based radioligands are widely used in preclinical and clinical PET studies because they bind with high affinity and selectivity to various receptors that are overexpressed on tumor cells. [17] Chelator-conjugated small peptide derivatives usually possess rapid tumor penetration, as well as fast clearance, from healthy tissues and the bloodstream, resulting in an enhanced target-to-non-target ratio. Despite the advantages of peptides, their use has certain limitations in development of PET imaging and radionuclide therapy agents, such as their short biological half-life can cause moderate tumor accumulation and therefore low tumor-to-background ratio, which reduce the resolution of PET images [18]. The pharmacokinetic properties of radiopeptides can be improved by adding appropriate structural elements to the linker unit that may contribute to their clinical translation. Glycosylation of peptides is one effective approach to this purpose, which can increase tumor accumulation and decrease liver and intestinal uptake [18]. Another fruitful method to improve the in vivo performance of peptide-based radioligands is pegylation which also can minimize their non-target uptake, but does not reduce their receptor-binding affinity [19].

We planned to introduce a lactosamine derivative as a carbohydrate moiety into the radiotracer containing cNGR peptide. *N*-acetylactosamine and its derivatives bind to the galectin-3 protein [20]; therefore the aforementioned beneficial effect of glycosylation, the use of this carbohydrate molecule can provide an opportunity for the synthesis of a dual-targeting radiopharmaceutical [21] since galectin-3 is also overexpressed in various tumor cells [22], for example in melanoma [23]. The enhanced galectin-3 expression correlates with poor overall survival in colon cancer patients [24,25]. Use of dual-targeting radiopeptides allows higher tumor accumulation and imaging quality for PET imaging through binding to two different targets. [26]. To prolong the biological half-life of the radiopeptides, we also designed the pegylation of the radiotracer via building PEG4 unit into the molecule. In this paper we have described the synthesis and radiolabeling of lactosamine-containing cNGR based glycopeptides and their preclinical investigation. To the best of our knowledge, this study is the first report of the synthesis and in vivo evaluation of ^68^Ga-labeled NGR-based glycopeptides for determination of aminopeptidase N (APN/CD13) and galectin-3 expression with PET imaging.

## 2. Materials and Methods

### 2.1. General

All reagents and solvents were obtained from commercial suppliers and used without further purification. 2,2′,2″-(10-(1-carboxy-4-((4-isothiocyanatobenzyl)amino)-4-oxobutyl)-1,4,7,10-tetraazacyclododecane-1,4,7-triyl)triacetic acid (p-NCS-Bn-DOTAGA) chelating agent was purchased from Chematech (Dijon, France). cyclic Lysine-Asparagine-Glycine-Arginine-Glutamic acid (cKNGRE) peptide was purchased from CASLO ApS (Lyngby, Denmark). All other reagents were purchased from Sigma-Aldrich. LC-MS was performed using a Waters Acquity UPLC Iclass system. Mass spectra were recorded on a Waters Acquity UPLC Iclass system (Waters, Milford, USA). For the HPLC system HPLC-MS grade acetonitrile (ACN) and methanol (MeOH) (Fisher Solutions, El Cajon, USA) and deionized water (Milli-Q, 18.2 MΩcm^−1^) were used. Ultra-purified hydrochloric acid, NaOAc and water were purchased from Merck KGaA (Darmstadt, Germany). ^68^Ga radioisotope was obtained from a ^68^Ge/^68^Ga isotope generator (Eckert-Ziegler, GalliaPharm^®^, Berlin, Germany, eluent: 0.1 M u.p. HCl). Activity measurements were carried out with a CAPINTEC CRC-15PET dose calibrator and a Perkin Elmer Packard Cobra gamma counter (Llantrisant, UK). Semipreparative RP HPLC were performed on a Waters LC Module 1 HPLC using a Luna C18 (250 × 10 mm) column and a KNAUER HPLC system using a semi-preparative Supelco Discovery^®^ Bio Wide Pore C18 10 µm (150 × 10 mm) column, connected to UV detector. Analytical HPLC and radio-HPLC were conducted using a Waters 2695 Alliance HPLC system and a KNAUER HPLC system, connected to UV detector and ATOMKI CsI scintillation detector. Semipreparative RP HPLC was performed with solvent A: 0.1% HCOOH and with solvent B: ACN/H_2_O (95:5 *v/v*), but in the case of the purification of DOTAGA-cKNGRE (15) the eluent A: 0.1% trifluoroacetic acid (TFA) in water and eluent B: 0.1% TFA in ACN/H_2_O (95:5, *v/v*) were used. Analytical HPLC was performed using a Luna C18 3 µm (150 × 4.6 mm) column (solvent A: oxalic acid (0.01 M pH = 3) and solvent B: acetonitrile) for Waters 2695 Alliance HPLC system and a Halo C18 5 µm (100 mm × 3.0 mm) column for the KNAUER HPLC system (eluent A: 0.1% TFA in water and eluent B: 0.1% TFA in ACN/H_2_O (95:5, *v/v*)). Purification of labeled radiopharmaceuticals were carried out with the following chromatographic material: Oasis HLB 1cc cartridge (Waters Corporation, Milford, MA, USA). Human plasma was obtained from Sigma-Aldrich (Saint Louis, MO, USA).

### 2.2. Chemistry

#### 2.2.1. DOTAGA-LacN (**3**)

To a solution of 3-azido-propyl β-D-galactopyranosyl-(1→4)-(2-amino-2-deoxy)-β-D-glucopyranoside (**1**) (6 mg, 0.014 mmol) in 0.1 M sodium carbonate buffer (900 µL, pH = 9.5) and dimethyl sulfoxide (DMSO) (100 µL) was added p-NCS-Bn-DOTAGA chelator **2** (10 mg, 0.014 mmol). After stirring overnight at room temperature, the reaction mixture was diluted with water, frozen and lyophilized. The crude product was purified by semipreparative RP-HPLC. For semipreparative RP-HPLC, a Luna C18 10 µm (250 × 10 mm) column was eluted at flow rate 4 mL/min using the following solvents: solvent A: 0.1% HCOOH, solvent B: 95% acetonitrile, gradient: 0 min:100% A, 2 min: 100% A, 32 min: 100% B, 40 min: 100% B. Peaks were detected at 254 nm. The product was collected between 9.9 and 10.7 min, frozen and lyophilized to produce compound **3** (4.04 mg, 28%) as a white powder. HRMS ESI calcd for: C_42_H_66_N_1_O_19_S_1_, 1047.4304 [M+H]^+^ and 524.2191 [M+H]^2+^. Found: 1047.4395 [M+H]^+^ and 524.2252 [M+H]^2+^. (Supplementary Material Figures S1 and S5).

#### 2.2.2. DBCO-cKNGRE (**6**)

To a solution of cKNGRE peptide **4** (7.25 mg, 0.012 mmol) in DMSO (300 µL) were added diisopropylethylamine (DIPEA) (10.5 µL, 0.06 mmol) and dibenzocyclooctyne-N-hydroxysuccinimidyl ester (DBCO-NHS) **5** (5 mg, 0.012 mmol). The reaction mixture was stirring overnight at room temperature. Then the reaction mixture was diluted with water, frozen and lyophilized., and the crude product was purified by semipreparative RP-HPLC.The conditions of semipreparative RP-HPLC were the same as for compound **3**. The product was collected between 18.7 and 19.3 min, frozen and lyophilized to give compound **6** (5 mg, 48%) as a white powder. HRMS ESI calcd for: C_42_H_54_N_12_O_9_, 872.4293 [M+2H]^+^ and 436.7186 [M+2H]^2+^. Found: 872.4024 [M+2H]^+^, 436.7118 [M+2H]^2+^.

#### 2.2.3. DBCO-PEG_4_-cKNGRE (**8**)

To a solution of cKNGRE peptide **4** (4.48 mg, 0.0077 mmol) in DMSO (300 µL) were added DIPEA (6.8 µL, 0.0385 mmol) and dibenzocyclooctyne-PEG4-N-hydroxysuccinimidyl ester (DBCO-PEG_4_-NHS) **7** (5 mg, 0.0077 mmol). The reaction mixture was stirring overnight at room temperature. Then the reaction mixture was dilute with water, frozen and lyophilized., and the crude product was purified by semipreparative RP-HPLC. The conditions of semipreparative RP-HPLC were the same as for compound **3**. The product was collected between 14.2 and 15 min, frozen and lyophilized to give compound **8** (2 mg, 23.24%) as a white powder. HRMS ESI calcd for: C_53_H_75_N_13_O_14_, 1119.5713 [M+2H]^+^ and 560.2896 [M+2H]^2+^. Found: 1119.5415 [M+2H]^+^, 560.2762 [M+2H]^2+^.

#### 2.2.4. DOTAGA-LacN-cKNGRE (**9**)

To a solution of compound **3** (3.5 mg, 0.0033 mmol) in DMSO (200 µL) was added a solution of compound **6** (2.5 mg, 0.00287 mmol) in DMSO (300 µL). After stirring overnight at room temperature, the reaction mixture was diluted with water, frozen and lyophilized. The crude product was purified by semipreparative RP-HPLC. The conditions of semipreparative RP-HPLC were the same as for compound **3**. The product was collected between 13.6 and 14.5 min, frozen and lyophilized to give compound **9** (1 mg, 18%) as a white powder. HRMS ESI calcd for: C_84_H_120_N_22_O_28_S_1_, 959.9274 [M+H]^2+^ and 640.6216 [M+H]^3+^. Found: 959.9204 [M+H]^2+^ and 640.6193 [M+H]^3+^. (Supplementary Material Figures S2 and S6).

#### 2.2.5. DOTAGA-LacN-PEG_4_-cKNGRE (**10**)

To a solution of compound **3** (1 mg, 0.95 µmol) in DMSO (200 µL) was added a solution of compound **8** (1 mg, 0.89 µmol) in DMSO (300 µL). After stirring overnight at room temperature, the reaction mixture was diluted with water, frozen and lyophilized. The crude product was purified by semipreparative RP-HPLC. The conditions of semipreparative RP-HPLC were the same as for compound **3**. The product was collected between 13.2 and 14.2 min, frozen and lyophilized to give compound **10** (1 mg, 52%) as a white powder. HRMS ESI calcd for: C_95_H_141_N_23_O_33_S_1_, 722.6682 [M+H]^3+^ and 542.2531 [M+H]^4+^. Found: 722.6637 [M+H]^3+^ and 542.2535 [M+H]^4+^. (Supplementary Material Figures S3 and S7).

#### 2.2.6. DOTAGA-cKNGRE (**11**)

To a solution of compound **2** (7.2 mg, 11.5 µmol) and compound **4** (5.8 mg, 9.95 µmol) in dimethylformamide 750 µL was added to 6.5 µL triethylamine (TEA) and 250 µL water. After stirring overnight at room temperature, the reaction mixture was diluted with water, frozen and lyophilized. The crude product was purified by semipreparative RP-HPLC. The conditions of semipreparative RP-HPLC were the following: the Supelco Discovery^®^ Bio Wide Pore C18 column 10 µm (150 mm × 10mm) was eluted at a flow rate 4 mL/min. Linear gradient elution (0 min 10% B; 5 min 10% B; 30 min 90% B) was used with eluent A: 0.1% TFA in water and eluent B: 0.1% TFA in ACN/H_2_O (95:5, *v/v*). Peaks were detected at 254 nm. The product was collected between 11.1 and 11.5 min, frozen and lyophilized to give compound **11** (6.6 mg, 55%) as a white powder. HRMS ESI calcd for: C_50_H_79_N_17_O_16_S_1_, 603.7884 [M+H]^2+^ and 402.8615 [M+H]^3+^. Found: 603.7916 [M+H]^2+^ and 402.8632 [M+H]^3+^. (Supplementary Material Figures S4 and S8).

### 2.3. Radiochemistry

#### 2.3.1. ^68^Ga Labeling of DOTAGA-LacN (**3**), DOTAGA-LacN-cKNGRE (**9**) and DOTAGA-LacN-PEG_4_-cKNGRE (**10**)

A ^68^Ge/^68^Ga-generator (Eckert-Ziegler, GalliaPharm^®^) was eluted with 0.1 M aq. ultra-purified hydrochloric acid. A volume of 1000 µL of ^68^Ga eluate (~80 MBq) was transformed into an Eppendorf vial, then 200 µL of NH_4_OAc (3 M, pH = 4) and 20 µL of aq. stock solution of compound **3**, compound **9**, and compound **10** (1 mg/mL) were added to, respectively. The reaction was performed at 95°C for 15 min. Then the reaction mixture was passed through a pre-conditioned (5 mL EtOH, 10 mL water) SPE cartridge (Oasis HLB 1cc). After washing of the cartridge with 1 mL of water, the radiolabeled product was eluted with 1:1 mixture of ethanol and saline (2 × 250 µL). The obtained solution of the labeled compounds was concentrated with stream of nitrogen, then the residue was diluted with 100 µL saline. For quality control, the radiochemical purity of the radiotracers was investigated by radio-HPLC on Alliance module with Luna C18 3 µm column. Solvent A: 0.1% oxalic acid, solvent B: 95% acetonitrile, gradient: 0 min: 100% A, 1 min: 100% A, 10 min: 100% B, 11 min: 100% B, 12 min: 100% A. The radiochemical purity was found to be better than 95% for all labeled compounds and the molar activity was 3.02 GBq/µmol ± 0.23 for [^68^Ga]-**3**, 4.69 GBq/µmol ± 0.15 for [^68^Ga]-**9** and 4.33 GBq/µmol ± 0.16 for [^68^Ga]-**10**. (Supplementary Material Figures S9–S11).

#### 2.3.2. ^68^Ga Labeling of DOTAGA-cKNGRE (**11**)

A ^68^Ge/^68^Ga-generator (Eckert-Ziegler, GalliaPharm^®^) was eluted with 0.1 M aq. ultra-purified hydrochloric acid. A volume of 1000 µL of ^68^Ga eluate was transformed into an Eppendorf vial, then 160 µL of NaOAc (1 M) and 3 µL of aq. solution of compound **11** (6 mM) were added to. The reaction was performed at 95°C for 5 min. Then the reaction mixture was passed through a pre-conditioned (5 mL EtOH, 10 mL water) SPE cartridge (Oasis HLB 1cc). After washing of the cartridge with 1 mL of water, the radiolabeled product was eluted with 2:1 mixture of ethanol and saline (300 µL). For quality control, the radiochemical purity of the radiotracer was investigated by radio-HPLC on KNAUER HPLC system with an analytical Halo C18 5 µm column (100 mm × 3.0 mm) and 0.7 mL/min flow rate. Eluent A: 0.1% TFA in water and eluent B: 0.1% TFA in ACN/H_2_O (95:5, *v/v*), gradient: 0 min 4% B; 2 min 4% B; 5 min 10% B; 10 min 30% B; 13 min 45% B. [^68^Ga]-11 was produced with high molar activity (8.51 ± 0.09 GBq/µmol) and with good radiochemical purity was better than 95%, in all cases. (Supplementary Material Figure S12).

#### 2.3.3. Determination of logP Value of ^[68^Ga]-**3**, [^68^Ga]-**9**, [^68^Ga]-**10** and [^68^Ga]-**11**

A volume of 50 µL of the purified [^68^Ga]-**3**, [^68^Ga]-**9**, [^68^Ga]-**10** and [^68^Ga]-**11** solution (2–3 MBq) was mixed in 450 µL of water and 500 µL of 1-octanol in an Eppendorf vial, respectively. The mixture was shaken with a vortex shaker for 5 min and centrifugated (9000 rpm) for 5 min. A 3 × 20 µL amount of both solvents was pipetted into a vial. The radioactivity of the fractions was determined with a gamma counter.

#### 2.3.4. Determination of In Vitro Stability of [^68^Ga]-**3**, [^68^Ga]-**9**, [^68^Ga]-**10** and [^68^Ga]-**11**

A volume of 50 µL of purified radiotracer solution (4–5 MBq) was added to 50 µL of human serum, oxalic acid (0.01 M) and ethylenediaminetetraacetic acid disodium salt dihydrate (Na_2_EDTA) (0.01 M) solution, respectively. Samples were analyzed at 0 min and after 60 and 120 min by radio-HPLC. The analytical conditions were the same as those used for quality control of the labeled complexes. (Supplementary Material Figures S13–S16).

### 2.4. Biology

#### 2.4.1. Animal Housing

12-week-old male C57BL/6 (n = 20) mice were housed in IVC system (Techniplast, Akronom Ltd., Budapest, Hungary) at 26 ± 2 °C with 55 ± 10% humidity and artificial lighting with a circadian cycle of 12 h. Semi-synthetic diet (SDS VRF-1, Animalab Ltd., Naszály út, Hungary) and tap water were available ad libitum to all the animals. The animals received human care and the experiments were authorized by the Ethical Committee for Animal Research, University of Debrecen, Hungary. Experimental animals were kept and treated in compliance with all applicable sections of the Hungarian Laws and directions and regulations of the European Union.

#### 2.4.2. B16-F10 Tumor Induction

For subcutaneous B16-F10 (mouse melanoma) tumor induction, C57BL/6 mice (n = 20) were anaesthetized with 3% isoflurane (Forane, AbbVie) using a dedicated small animal anesthesia device (Tec3 Isoflurane Vaporizer, Eickemeyer, UK) and mice were injected subcutaneously with 3 × 10^6^ B16-F10 cells in 150 µL of sterile saline into the left shoulder area of the animals. In vivo and ex vivo experiments were carried out 10 ± 1 days after subcutaneous injection of tumor cells at the tumor volume of 80 ± 2 mm^3^.

#### 2.4.3. In Vivo PET Imaging and Image Analysis

For in vivo biodistribution studies B16-F10 tumor-bearing mice were anaesthetized with 3% isoflurane and were injected with 6.6 ± 0.3 MBq [^68^Ga]-**11**, or [^68^Ga]-**3**, or [^68^Ga]-**9** or [^68^Ga]-**10** in 150 μL saline via the lateral tail vein 10 ± 1 days after subcutaneous injection of tumor cells. 20 min static PET scans were performed 70 min after intravenous injection of the radiotracers from the tumorous area using the preclinical miniPET-II device (University of Debrecen, Faculty of Medicine, Department of Medical Imaging, Division of Nuclear Medicine and Translational Imaging). After image reconstruction, volumes of interest (VOIs) were manually drawn around the examined regions using the BrainCAD image analysis software and quantitative standardized uptake values (SUVs) values were calculated.

#### 2.4.4. Statistical Analysis

Data were presented as mean ± SD. The significance was calculated using Student’s *t*-test (two-tailed), two-way ANOVA, and Mann-Whitney U-test. The significance level was set at *p* ≤ 0.05 unless otherwise indicated.

## 3. Results

### 3.1. Chemistry

**Scheme 1:**
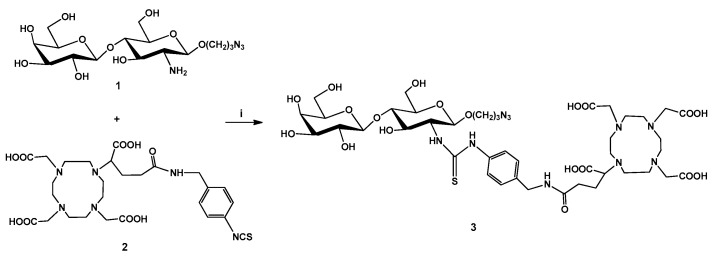
Reagents and conditions: (**i**) Na_2_CO_3_ buffer (0.1 M, pH 9.5); DMSO, rt, 24 h, 28%.

First, we accomplished the synthesis of chelator-conjugated lactosamine derivative **3**. 3-Azido-propyl-β-D-galactopyranosyl-(1→4)-(2-amino-2-deoxy)-β-D-glucopyranoside (**1**)was coupled with p-NCS-Bn-DOTA-GA (**2)** in DMSO and in 0.1 M sodium carbonate buffer which resulted in compound **3**. (Scheme 1). The detailed synthesis of compound **1** is described in the Supplementary Material Part 1. For the synthesis of compound **1**, thiophenyl (2,3,4,6-tetra-*O*-acetyl-β-D-galactopyranosyl)-(1→4)-(3,6-di-*O*-acetyl-2-N-trichloroacetyl-2-deoxy-β-D-glucopyranoside (**I**) [27] was chosen as a starting material which was provided by Dr. István Bajza (GlycOptim Ltd.). The compound **3** was radiolabeled to investigate the diagnostic potential of this carbohydrate derivatives for the detection of galectin-3 expression.

**Scheme 2:**
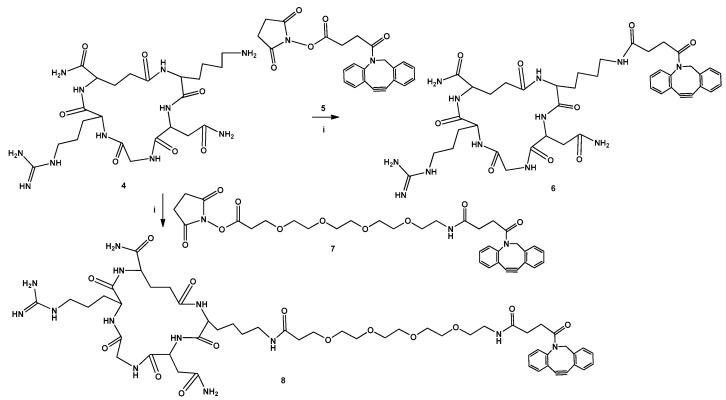
Reagents and conditions: (**i**) DIPEA; DMSO, rt, 24 h, 48% for compound **6** and 23.2% for compound **8**.

Since the conjugation of the peptide to chelator-bearing lactosamine **3** was designed with copper-free click reaction, the cKNGRE (**4**) was functionalized with cyclooctyne moiety using DBCO-NHS (**5**) in DMSO and in the presence of diisopropylethylamine (DIPEA) to give compound **6**. For the synthesis of PEG_4_-linker-containing radiotracer the peptide **4** was reacted with DBCO-PEG_4_-NHS (**7**) in the same way as described above to yield compound **8**. (Scheme 2).

**Scheme 3:**
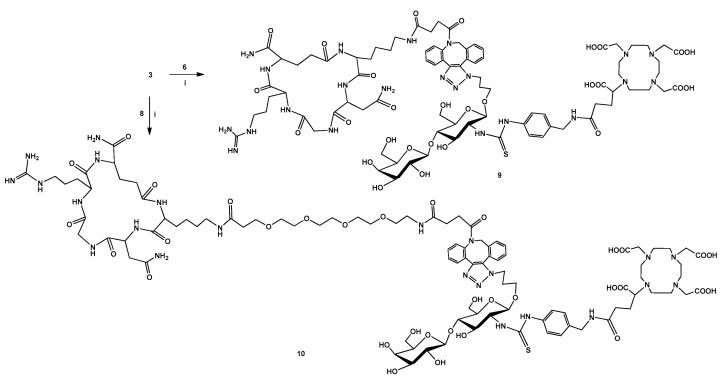
Reagents and conditions: (**i**) DMSO, rt, 24 h, 18% for compound **9** and 52% for compound **10**.

Subsequently, compound **6** and compound **8** were conjugated to chelator-bearing lactosamine **3** with a strain promoted click reaction in DMSO, respectively (Scheme 3).

**Scheme 4:**
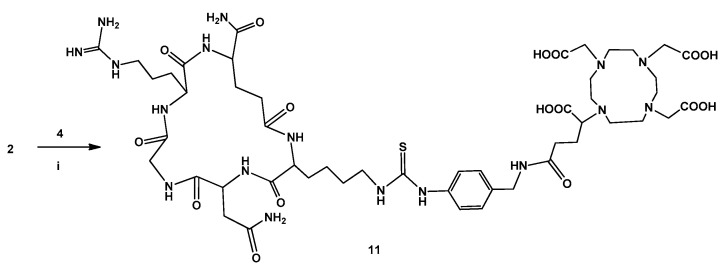
Reagents and conditions: (**i**) TEA, DMF-H_2_O (3:1), rt, 24 h, 55%.

To investigate the effect of the structural changing on the in vivo performance of the radioglycopeptides we carried out the synthesis of the DOTAGA-cKNGRE (**11**) as a reference. Thus, p-NCS-Bn-DOTA-GA (**2**) was coupled with cKNGRE (**4**) in the presence of triethylamine in dimethylformamide and water (Scheme 4).

The glycopeptides (**9** and **10**) and compound **11** are suitable precursors for radiolabeling with ^68^Ga radioisotope. The synthetized compounds were characterized by analytical RP-HPLC and HRMS ESI.

### 3.2. Radiochemistry

Radiolabeling of the synthetized precursors (**3**, **9**, **10**, and **11**) was performed using ^68^Ge/^68^Ga-generator produced ^68^Ga isotope with high radiochemical purity (>95%). The labeled complexes were purified with solid phase extraction (SPE) using Oasis HLB 1cc cartridge. The radiochemical purity of the obtained radiopharmaceuticals was analyzed by radio-HPLC (>95%). The octanol/water partition coefficients (log*P*) of ^68^Ga-labeled radioligands were determined. The log*P* value was found to be −3.34 for [^68^Ga]-**3**, −3.68 for [^68^Ga]-**9**, −3.69 for [^68^Ga]-**10** and −4.13 for [^68^Ga]-**11**. According to the low log*P* values, the radiolabeled compounds were hydrophilic.

Furthermore, the stability of the labeled ligands was investigated; therefore, the solution of the radiotracers was mixed with a solution of human serum, oxalic acid (0.01 M) and Na_2_EDTA (0.01 M) at room temperature, respectively. Samples were then taken at various time points (0, 60, 120 min) and analyzed by radio-HPLC. Radiochromatograms showed that none of the radiotracers was degraded at the time points studied, as the radiochemical purity of all samples was >95%.

### 3.3. Biology

**Figure 1:**
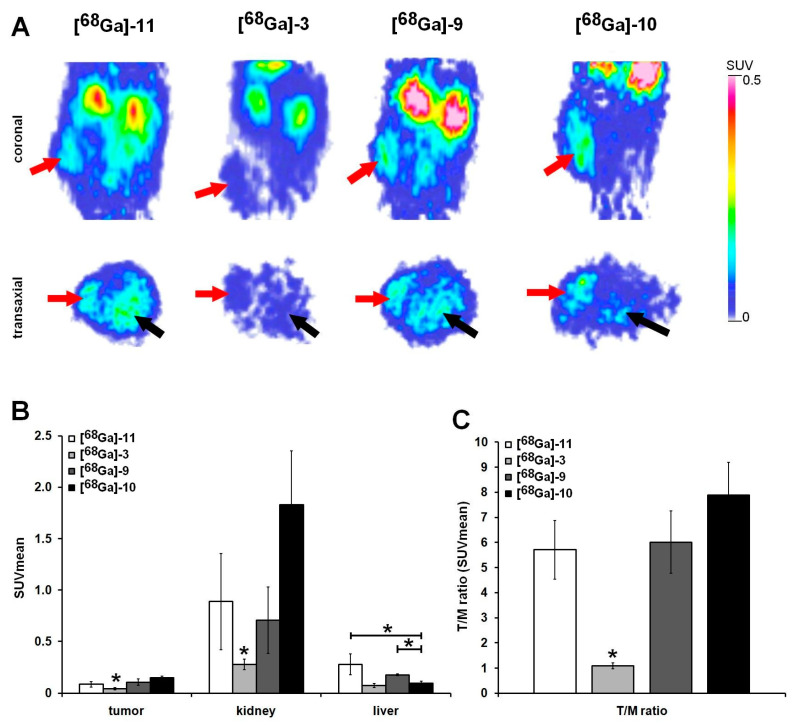
In vivo PET imaging of [^68^Ga]-**11**, [^68^Ga]-**3**, [^68^Ga]-**9** and [^68^Ga]-10 accumulation in B16-F10 tumor-bearing mice 10 ± 1 days after tumor cell inoculation and 70 min after intravenous injection of the radiotracers (**A** panel). Red arrows: B16-F10 tumor; black arrows: liver. (**B**,**C**) panels: quantitative SUVmean and tumor-to-background (muscle) ratio (T/M) analysis of tracer uptake in mice (n = 3/radiopharmaceutical) 70 min post-injection. Significance level: *p* ≤ 0.05 (*). SUV: standardized uptake value; T/M: tumor-to-muscle ratio. SUV values are presented as mean ± SD.

PET imaging was accomplished with [^68^Ga]-**11**, [^68^Ga]-**3**, [^68^Ga]-**9**, and [^68^Ga]-**10** using B16-F10 melanoma tumor-bearing mice. Representative decay-corrected PET images are shown in Figure 1 (A panel). Among the investigated radiotracers the subcutaneously growing B16-F10 tumors were clearly identified using [^68^Ga]-**11**, [^68^Ga]-**9** and [^68^Ga]-**10**, in contrast, moderate accumulation was observed in the tumors after the injection of [^68^Ga]-**3**. This observation was confirmed by the quantitative SUVmean and T/M analysis (Figure 1B,C panels), where we found significantly (*p* ≤ 0.05) lower [^68^Ga]-**3** (SUVmean: 0.04 ± 0.01; T/M ratio: 1.09 ± 0.12) accumulation than that of the [^68^Ga]-**11** (SUVmean: 0.09 ± 0.03; T/M ratio: 5.71 ± 1.17), [^68^Ga]-**9** (SUVmean: 0.11 ± 0.03; T/M ratio: 6.01 ± 1.24) and [^68^Ga]-**10** (SUVmean: 0.15 ± 0.01; T/M ratio: 7.89 ± 1.31) radiotracers. However, there were differences between the accumulations of the radiotracers in the liver and kidney (Figure 1B panel).

Furthermore, despite the APN/CD13 specificity, the uptake was significantly (*p* ≤ 0.05) lower for the PEG-containing [^68^Ga]-**10** (liver SUVmean: 0.10 ± 0.02) than for the [^68^Ga]-**11** (liver SUVmean: 0.28 ± 0.10) and [^68^Ga]-**9** (liver SUVmean: 0.18 ± 0.01) probes.

**Figure 2:**
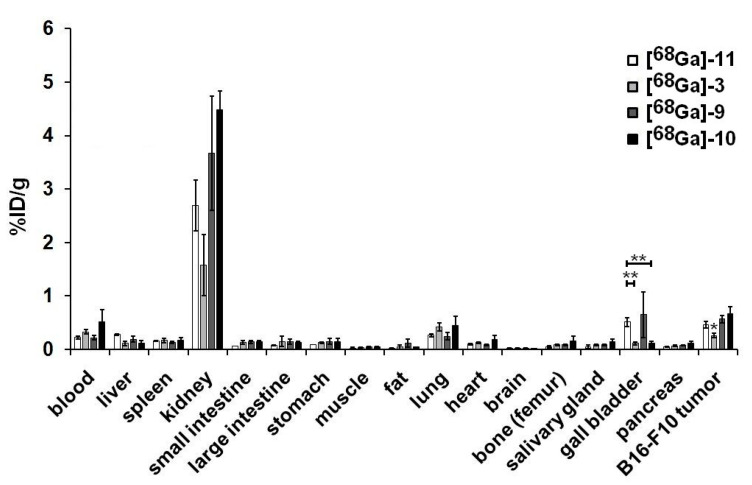
Ex vivo biodistribution studies. Ex vivo assessment of the accumulation of [^68^Ga]-**11**, [^68^Ga]-**3**, [^68^Ga]-**9**, and [^68^Ga]-**10** in experimental subcutaneous B16-F10 tumors 90 min after tracer injection and 10 ± 1 days after tumor cell inoculation. %ID/g tissue values are presented as mean ± SD. Significance levels: *p* ≤ 0.05 (*) and *p* ≤ 0.01 (**).

In vivo PET imaging results correlated well with the ex vivo data shown in Figure 2.

## 4. Diccussion

Numerous studies demonstrated with blocking experiments that NGR peptide analogs bind to APN/CD13 receptor with high selectivity, making them excellent vector molecules for the synthesis of peptide-targeted radiopharmaceuticals [13,14,15,16]. ^68^Ga-labeling of an NGR derivative conjugated with a bifunctional chelator can provide a PET imaging agent for the detection of APN/CD13-positive tumor cells. For example, to detect APN/CD13 expression, ^68^Ga-DOTA-NGR was prepared by Zang et al. Based on MicroPET imaging, high tumor uptake of this radiotracer was observed in the tumor site in A549 tumor xenografts [13]. Furthermore, Chen et al. described the synthesis and preclinical evaluation of two radioligands containing monomeric and dimeric NGR peptides which were radiolabeled with ^64^Cu PET isotope (β^+^ 17.8%; t_1/2_ = 12.7 h; E(β^+^) = 655 keV) [14]. In 2015 Máté et al. reported the preparation of ^68^Ga-NOTA-c(NGR) and comparison of its in vivo performance with α_v_β_3_ integrin selective ^68^Ga-NODAGA-[c(RGD)]_2_. In rats bearing Ne/De tumors, ^68^Ga-NOTA-c(NGR) radiotracer showed greater accumulation in tumors than ^68^Ga-NODAGA-[c(RGD)]_2_ [15]. In another study [16] a c(NGR) analog modified by *N*-methylation (c[CH_2_-CO-Lys(NODAGA)-Asn-N(Me)Gly-Arg-Cys]-NH_2_) to prevent asparagine deamidation, was synthesized and radiolabeled with ^68^Ga isotope. The pharmacokinetic properties of this radiopeptide were compared with the ^68^Ga-labeled non-*N*-methylated version (c[CH_2_-CO-Lys(NODAGA)-Asn-Gly-Arg-Cys]-NH_2_) and two other cNGR-based radiotracers, the NOTA-c(NGR) [15] and NODAGA-c(NGR). All labeled compounds showed high tumor accumulation in APN/CD13-positive He/De and Ne/De tumors. These studies confirmed that chelator-conjugated NGR analogs are promising PET agents for APN-overexpressing tumors.

The design of the radiotracer, including the chemical structure of the targeting molecule, the chelating agent, and the linker moiety, has a remarkable effect on the biological efficacy of radiolabeled compounds [18,19]. One effective method to improve the in vivo performance of a peptide-targeted radiotracer is glycosylation, because glycosylated peptides are more hydrophilic than the original peptides, which increases their solubility and bioavailability, as well as their enzymatic stability with the endogenous proteases [18]. We chose a lactosamine derivative as a carbohydrate moiety for glycosylation of a NGR derivatives hence it is natural ligand of galectin-3 receptor which is overexpressed in numerous tumors [22]. This receptor is a carbohydrate-binding protein and can recognize the β-galactoside motif of glycoconjugates [28]. It is involved in different pathological processes during cancer progression, such as cell adhesion and migration, proliferation, differentiation, angiogenesis and metastasis [29]. Therefore, the incorporation of lactosamine moiety into a DOTAGA-conjugated NGR derivetive can result in dual-targeting radiopharmaceutical. Dual-targeting approach is a relatively new method in the field of PET imaging, but previously numerous bispecific antibodies were developed for therapeutic applications [30]. Pegylation can also improve the pharmacokinetic properties of the radiopeptides [19]. To investigate the effect of glycosylation and pegylation on the in vivo properties of a radiotracer, three NGR-based radiopeptides were synthesized. We have previously reported the synthesis and radiochemical investigation of a glycopeptide, ^68^Ga-NODAGA-LacN-E[c(RGDfK)]_2_, which may be suitable for the detection of α_v_β_3_ integrin and galectin-3 expression in tumor endothelial and cancer cells by PET imaging [31]. In this report, a similar synthetic approach was used to prepare NGR-based radioligands as previously applied for ^68^Ga-NODAGA-LacN-E[c(RGDfK)]_2_. In this case, however, a DOTAGA derivative was chosen to complex the gallium-68 radioisotope hence DOTA-based compounds are excellently chelate of various diagnostic radiometals, such as gallium-68, scandium-44 and copper-64, as well as various therapeutic metals, e.g., lutetium-177 and bismuth-213. Thus, for targeted radionuclide therapy the newly synthetized DOTAGA-conjugated peptides may also be labeled with therapeutic radiometals.

The same lactosamine derivative, namely 3-azido-propyl-β-D-galactopyranosyl-(1→4)-(2-amino-2-deoxy)-β-D-glucopyranoside (**1**) was applied to introduce carbohydrate moiety into the radiotracer as used in the synthesis of ^68^Ga-NODAGA-LacN-E[c(RGDfK)]_2_. However, for the preparation of this compound another synthetic route has been developed (See Supplementary Material Part 1). The azidopropyl aglycone of the compound **1** allows the conjugation to peptides via a click reaction.

After successful preparation of the NGR- and/or lactosamine-containing precursors (**3**, **9**, **10** and **11**), their ^68^Ga labeling was performed with high radiochemical purity using conventional procedure. The octanol/water partition coefficients were also determined and similarly low log*P* values indicated that the radiolabeled compounds were hydrophilic. Interestingly, the log*P* of the non-glycosylated radiopeptide was lower than that of the glycosylated radiopeptides. The DBCO moiety formed during bioconjugation is likely to compensate for the hydrophilicity of the lactosamine residue. In addition, in vitro stability tests of radiolabeled compounds in human serum have been performed and remained intact for two hours.

Previous studies already reported that B16-F10 melanoma tumors showed strong positivity for APN/CD13 and galectin-3 receptors [23,32,33]. For the determination of receptor targeting potential and biodistribution of the [^68^Ga]-**11**, [^68^Ga]-**3**, [^68^Ga]-**9**, and [^68^Ga]-**10** radiotracers in vivo PET imaging studies and ex vivo tissue distribution studies were performed using B16-F10 melanoma tumor-bearing mice. 10 ± 1 days after tumor cell inoculation, the B16-F10 tumors were clearly visualized using [^68^Ga]-**11**, [^68^Ga]-**9**, and [^68^Ga]-**10** probes, in contrast, significantly lower uptake was observed after the injection of [^68^Ga]-**3** (Figure 1A panel). This finding was confirmed by the quantitative image analysis (Figure 1B,C panels). These results suggest the strong APN/CD13 positivity of the investigated B16-F10 melanoma tumors and high affinity of the cKNGRE-containing radiotracers for this receptor.

By analyzing the liver and kidney uptake, differences were observed between the radiotracers (Figure 1). This finding shows the route of excretion from the body according to the log*P* values (approximately −3.5); moreover, the accumulation of kidney and liver is greatly influenced by the expression of galectin-3 and APN/CD13 in these tissues. It is known from the literature that the APN/CD13 expression is high in the kidney and liver [33], in addition, low galectin-3 expression was found in the healthy kidney and liver tissues [34,35,36]. Consistent with these observations, we also found higher liver and kidney accumulation using the APN/CD13-specific cKNGRE-containing radiotracers than that of the galectin-3-specific [^68^Ga]-**3** probe. Moreover, the accumulation in the liver was significantly (*p* ≤ 0.05) lower for the PEG-containing [^68^Ga]-**10** than for the [^68^Ga]-**3,** [^68^Ga]-**11** and [^68^Ga]-**9** due to pegylated proteins increase the circulation time in the body and decrease immunogenicity [37]. The prolonged circulation time was also confirmed by our ex vivo study in which the blood showed higher radioactivity concentration for [^68^Ga]-**10** 90 min after the injection, compared to the other investigated molecules (Figure 2). Our in vivo PET imaging results correlated well with the ex vivo data shown in Figure 2.

## 5. Conclusions

We have successfully completed the synthesis and radiolabeling of two NGR-based glycopeptides (**9** and **10**) and DOTAGA-cKNGRE (**11**), which are suitable for the detection of APN-positive tumors by PET imaging. The in vivo properties of the novel radioligands were compared by PET imaging and ex vivo biodistribution. These studies showed that all three NGR-based radiopeptides were able to visualize APN/CD13 receptor expression in B16-F10 melanoma tumors by PET imaging. The glycosylation and pegylation of NGR derivatives resulted in slightly enhanced tumor uptake. However, for the [^68^Ga]-**10** tracer, liver uptake was significantly lower and tumor-to-background (muscle) ratio (T/M) was higher due to pegylation and glycosylation, resulting in a more contrasting PET image. These pharmacokinetic properties are advantageous for potential radiotherapeutic use if the precursor molecule **10** is labeled with a therapeutic radiometal. In addition, the suitability of [^68^Ga]-**3** for the determination of galectin-3 levels was investigated, but the use of this radiotracer resulted in moderate tumor uptake, which may be explained by the fact that this lactosamine derivative is not a sufficiently specific ligand for galactin-3 receptor. Presumably, therefore, the synergetic effect of the dual-targeting approach was also not significant. However, the application of pegylation and glycosylation of the NGR derivative improved the pharmacokinetic characteristics of the radiopeptide.

## Data Availability

Not applicable.

## Supplementary Materials

The following are available online at https://www.mdpi.com/article/10.3390/pharmaceutics13122103/s1. Scheme S1: Synthesis of compound **1**, Figure S1: Analytical RP-HPLC chromatograms of compound **3**, Figure S2: Analytical RP-HPLC chromatograms of compound **9**, Figure S3: Analytical RP-HPLC chromatograms of compound **10**, Figure S4: Analytical RP-HPLC chromatograms of compound **11**, Figure S5: Mass spectrum of compound **3**, Figure S6: Mass spectrum of compound **9**, Figure S7: Mass spectrum of compound **10**, Figure S8: Mass spectrum of compound **11**, Figure S9: UV-and radio-HPLC chromatograms of [^68^Ga]-**3**, Figure S10: UV-and radio-HPLC chromatograms of [^68^Ga]-**9**, Figure S11: UV-and radio-HPLC chromatograms of [^68^Ga]-**10**, Figure S12: UV-and radio-HPLC chromatograms of [^68^Ga]-**11**, Figure S13: Radio-HPLC chromatograms of serum stability test of [^68^Ga]-**3**, Figure S14: Radio-HPLC chromatograms of serum stability test of [^68^Ga]-**9**, Figure S15: Radio-HPLC chromatograms of serum stability test of [^68^Ga]-**10**, Figure S16: Radio-HPLC chromatograms of serum stability test of [^68^Ga]-**11**. Ex-vivo biodistribution studies.
