# Supplementary material for: Synthesis of 68Ga-Labeled cNGR-Based Glycopeptides and In Vivo Evaluation by PET Imaging
Source: Pharmaceutics. 2021 Dec 7;13(12):2103. doi: 10.3390/pharmaceutics13122103 (PMC8703807; doi:10.3390/pharmaceutics13122103)
Supplement: Supplementary file 1 [file pharmaceutics-13-02103-s001.zip › pharmaceutics-1474001-sup.pdf]

pharmaceutics

MDPI

Supplementary Materials: Synthesis of ^{68}Ga-Labeled cNGR-Based Glycopeptides and InVivo Evaluation by PET Imaging

Barbara Gyuricza, Judit P. Szabó, Viktória Arató, Noémi Dénes, Ágnes Szűcs, Katalin Berta, Adrienn Kis, Dániel Szücs, Viktória Forgács, Dezső Szikra, István Kertész, György Trencsényi, Anikó Fekete

Part 1: Synthesis of 3-azido-propyl-β-D-galactopyranosyl-(1→4)-(2-amino-2-deoxy)-β-D-glucopyranoside (1)

For this synthesis, thiophenyl (2,3,4,6-tetra-*O*-acetyl-β-D-galactopyranosyl)-(1→4)-(3,6-di-*O*-acetyl-2-*N*-trichloroacetyl-2-deoxy-β-D-glucopyranoside (**I**) [37] was chosen as a starting material which was provided by Dr. István Bajza (GlycOptim Ltd.). Compound **I** was glycosylated with bromo propanol in the presence of *N*-iodosuccinimide/triflic acid (NIS/TfOH) promotor system and then was converted to azido-propyl glycoside by nucleophilic substitution to yield compound **II**. After Zemplén de-*O*-acetylation the *N*-trichloroacetyl group was removed by basic hydrolysis from compound **IV** to yield compound **1** as a starting material of the synthesis of NGR-based glycopeptides (Scheme S1.)

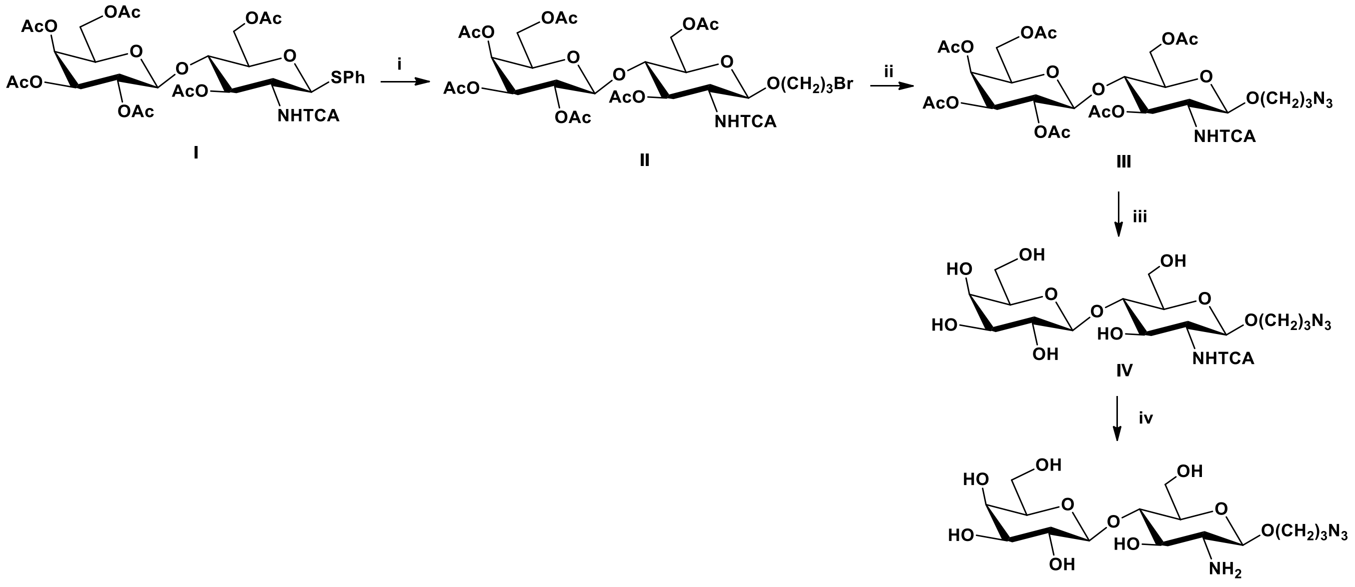

Scheme S1. Synthesis of compound 1. Reagents and conditions: (i) $Br(CH_2)_3Br$; NIS; TfOH; THF; DKM, 0°C, 24 h, 76%; (ii) NaN_3; DMF, 60°C, 24 h, 64%; (iii) NaOMe; MeOH, rt, 24 h, 84%; (iv) NaOH, 56 %.

Materials and Methods:

General

All reagents and solvents were obtained from commercial suppliers and used without further purification. All other reagents were purchased from Sigma-Aldrich. TLC was performed on Kieselgel 60 F_{254} (E. Merck) with detection by UV detector and charring with 5 % sulphuric acid in ethanol. Column chromatography was performed on Silica gel 60 (Merck 63–200 mesh). The ^{1}H (400 MHz) and ^{13}C NMR (128 MHz) spectra were recorded with Bruker DRX-400 spectrometers. Internal references: TMS (0.000 ppm for ^{1}H), $CDCl_3$ (77.00 ppm for ^{13}C for organic solution). Mass spectra were recorded on a maXis II UHR ESI-QTOF MS Bruker instrument (Bruker, Billerica, USA).

3-Bromo-propyl (2,3,4,6-tetra-*O*-acetyl-β-D-galactopyranosyl-(1→4)-(3,6-di-*O*-acetyl-2-*N*-trichloroacetyl-2-deoxy-β-D-glucopyranoside (II)

To a solution of compound **I** (1 g, 1.2 mmol) in dry dichloromethane (10 mL) and 4 Å molecular sieves was added 3-bromo-propanol (220 µL, 1.2 mmol). The reaction mixture was cooled to 0 °C, and were added NIS (332 mg, 1.475 mmol) in THF (500 µL) and TfOH (13 µL, 0.145 mmol). After stirring over night the reaction mixture was neutralized by pyridine, diluted with CH_2Cl_2, washed with $Na_2S_2O_3$ (10%) and water, dried and evaporated to dryness *in vacuo* at 50 °C. R_f 0.25 (CH_2Cl_2 – EtOAc 8:2). The crude product was purified by column chromatography (Silica gel: 100 g, eluent: CH_2Cl_2 – EtOAc 8:2, fractions: 5 mL). The fractions were analyzed with TLC and the product-containing fractions were collected and evaporated to dryness *in vacuo* at 50 °C to yield **II** (782 mg, 76%) as a colorless syrup. ^{1}H NMR (400 MHz, Chloroform-d) δ 5.36 (d, J=3.3 Hz, 1H), 5.24 (t, J=9.7 Hz, 1H), 5.12-5.02 (m, 1H), 4.98 (dd, J=7.4, 3.3 Hz, 1H), 4.63-4.45 (m, 3H), 4.22-3.88 (m, 7H), 3.87-3.77 (m, 1H), 3.69-3.58 (m, 3H), 3.51-3.44 (m, 2H), 2.15, 2.14, 2.07, 2.06, 2.05, 1.97 (s, 18H). ^{13}C NMR (Chloroform-d) 170.90, 170.42, 170.37, 170.06, 169.13 and 162.28 (CO), 101.42 and 100.95 (C-1 and C-1′), 76.41, 72.89, 72,02, 70.92, 70.80, 69.10 and 66.70 (C-2′, C-3, C-3′, C-4, C-4′, C-5 and C-5′) 67.11 (CH_2) 62.00 and 60.88 (C-6, C-6′), 55.46 (C-2), 32.34 and 30.44 (CH_2), 21.09, 20.95, 20.83, 20.66 and 20.55 (CH_3CO); HRMS ESI calcd for $C_{29}H_{39}BrCl_3NNaO_{17}$, 880.0359 [M+Na]. Found: 880.0355 $[M+Na]^+$.

3-Azido-propyl (2,3,4,6-tetra-*O*-acetyl-β-D-galactopyranosyl-(1→4)-(3,6-di-*O*-acetyl-2-*N*-trichloroacetyl-2-deoxy-β-D-glucopyranoside (III)

To a solution of compound II (782 mg, 0.91 mmol) in dry DMF (10 mL) was added NaN_3 (296 mg, 4.55 mmol). After stirring over night at 60 °C, the reaction mixture was evaporated to dryness *in vacuo* at 50 °C, then diluted with CH_2Cl_2, washed with water and satd aq $NaHCO_3$, dried and evaporated to dryness *in vacuo* at 50 °C. R_f 0.22 (CH_2Cl_2 – EtOAc 8:2). The crude product purified by column chromatography (Silica gel: 65 g, eluent: CH_2Cl_2 – EtOAc 8:2, fractions: 5 mL). The fractions were analyzed with TLC and the product-containing fractions were collected and evaporated to dryness *in vacuo* at 50 °C to yield **III** (475 mg, 64%) as a colourless syrup. ^{1}H NMR (400 MHz, Chloroform-d) δ 5.36 (dd, J=3.5, 1.2 Hz, 1H), 5.23 (dd, J=10.5, 8.7 Hz, 1H), 5.09 (dd, J=10.4, 7.8 Hz, 1H), 4.98 (dd, J=10.4, 3.4 Hz, 1H), 3.67-3.50 (m, 2H), 3.37 (t, J=6.5 Hz, 2H), 2.14, 2.08, 2.07, 2.06 (6s, 18H), 1.91-1.73 (m, 2H). ^{13}C NMR (Chloroform-d): 170.91, 170.45, 170.42, 170.12, 170.10, 169.19 and 162.24 (CO), 101.42 and 100.80 (C-1 and C-1′), 76.33, 72.97,71.96, 70.95, 70.84, 69.14 and 66.72 (C-2′, C-3, C-3′, C-4, C-4′, C-5 and C-5′), 66.31 (CH_2) 62.02 and 60.91 (C-6 and C-6′), 55.48 (C-2), 47.93 and 29.03 (CH_2), 20.94, 20.83, 20.71, 20.68, 20.63 and 20.56 (CH_3CO); HRMS ESI calcd for: $C_{29}H_{39}Cl_3N_4NaO_{17}$, 843.1268 [M+Na]. Found: 843.1261 [M+Na]+.

3-Azido-propyl β-D-galactopyranosyl-(1→4)-(2-*N*-trichloroacetyl-2-deoxy)-β-D-glucopyranoside (IV)

To a solution of compound **III** (475 mg, 0.56 mmol) in CH_2Cl_2 (5 mL) and MeOH (10 mL) was added catalytic amount of NaOMe (pH 8-9). The reaction mixture was stirred overnight at room temperature, neutralized by AQ50W-X4 H^+ ion exchange resin, filtered and evaporated to dryness *in vacuo* at 50 °C. R_f 0.37 (CH_2Cl_2 – MeOH 8:2) The crude product was purified by column chromatography (Silica gel: 40 g, eluent: CH_2Cl_2-MeOH 85:15, fractions: 3 mL). The fractions were analyzed with TLC and the product-containing fractions were collected and evaporated to dryness *in vacuo* at 50 °C to yield **IV** (267 mg, 84%) as a colourless syrup. ^{1}H NMR (400 MHz, Methanol-d4): δ 4.56 (d, J=8.2 Hz, 1H), 4.39 (d, J=7.3 Hz, 1H), 3.99-3.90 (m, 2H), 3.87 (dd, J=12.1, 4.2 Hz, 1H), 3.84-3.62 (m, 5H), 3.61-3.28 (m, 8H), 1.86-1.73 (m, 2H). ^{13}C NMR (Methanol-d4): 164.36 (CO), 105.14 and 102.17 (C-1 and C-1′), 81.18, 77.17, 76.63, 74.85, 73.42, 72.67 and 70.38 (C-2′, C-3, C-3′, C-4, C-4′, C-5 and C-5′), 67.27 (CH_2) 62.61 and 61.90 (C-6, C-6′), 58.67 (C-2), 30.13 (CH_2); HRMS ESI calcd for: $C_{17}H_{27}Cl_3N_4NaO_{11}$, 591.0634 [M+Na]. Found: 591.0630 $[M+Na]^+$.

3-Azido-propyl β-D-galactopyranosyl-(1→4)-(2-amino-2-deoxy)-β-D-glucopyranoside (1)

Compound **IV** (40 mg, 0.0704 mmol) was dissolved in NaOH (1 M, 2 mL). After stirring overnight at room temperature the reaction mixture was neutralized with HCl (1 M, 2 mL), and evaporated to dryness *in vacuo* at 50 °C. The crude product was purified by size exclusion column chromatography (Sephadex® LH-20, eluent:methanol, fractions: 0.5 mL). The fractions were analyzed with TLC and the product-containing fractions were collected and evaporated to dryness *in vacuo* at 50 °C to yield **1** as a colourless syrup (17.7 mg, 56 %). The characterization of this compound was described [31].

Reference:

27. Bajza, I., Dekany, G., Agoston, K., Perez, I. F., Boutet, J., Hederos, M., Horvath, F., Kovacs-Penzes, P., Kroeger, L., Roeh-rig, C., Schroven, A., Vrasidas, I., Trinka, P., Kalmar, L., Kovacs, I., Demko, S., Agoston, A., Risinger, C. A method for preparation of the tetrasaccharide lacto-N-neotetraose (LNnt) containing N-acetyllactosamine. Patent: WO 2011100980 A1 (25.08.2011.)

31. Gyuricza, B.; Szabó, J.P.; Arató, V.; Szücs, D.; Vágner, A.; Szikra, D.; Fekete, A. Synthesis of Novel, Dual-Targeting ^{68}Ga-NODAGA-LacN-E[c(RGDfK)]$_2$ Glycopeptide as a PET Imaging Agent for Cancer Diagnosis. Pharmaceutics 2021, 13, 796.

Part 2: Analytical RP HPLC chromatograms of compound 3, 9, 10 and 11; mass spectra of compound 3, 9, 10 and 11; UV- and radio-HPLC chromatograms of [^{68}Ga]-3, [^{68}Ga]-9, [^{68}Ga]-10 and [^{68}Ga]-11, radio-HPLC chromatograms of serum stability test [^{68}Ga]-3, [^{68}Ga]-9, [^{68}Ga]-10 and [^{68}Ga]-11.

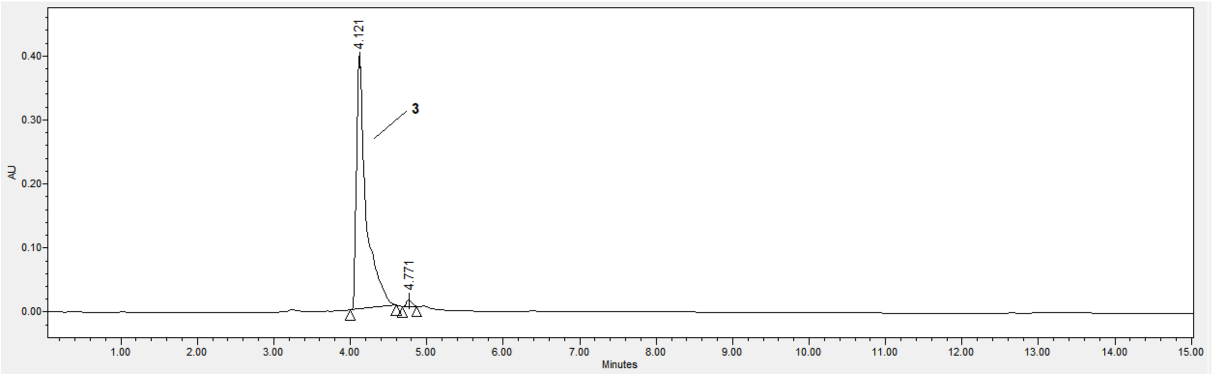

Figure S1 Analytical RP-HPLC chromatograms of compound **3.**

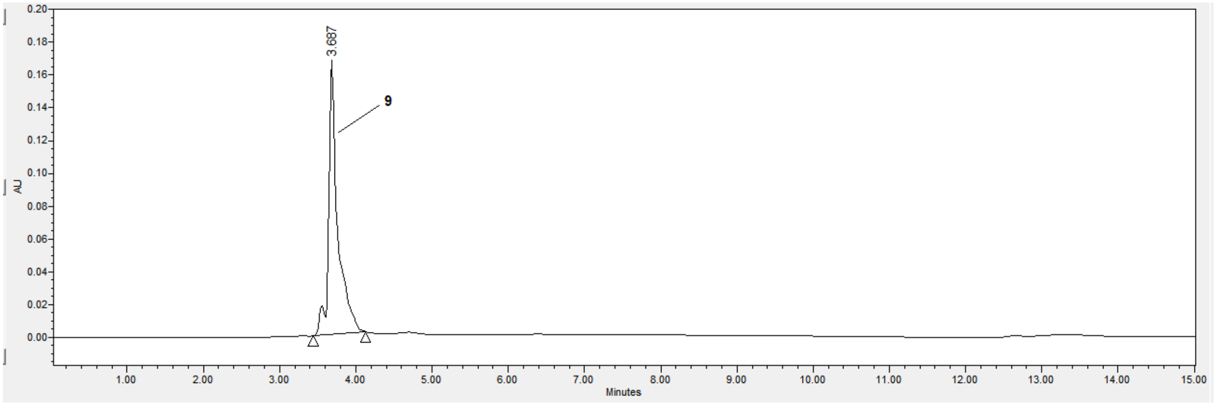

Figure S2 Analytical RP-HPLC chromatograms of compound **9.**

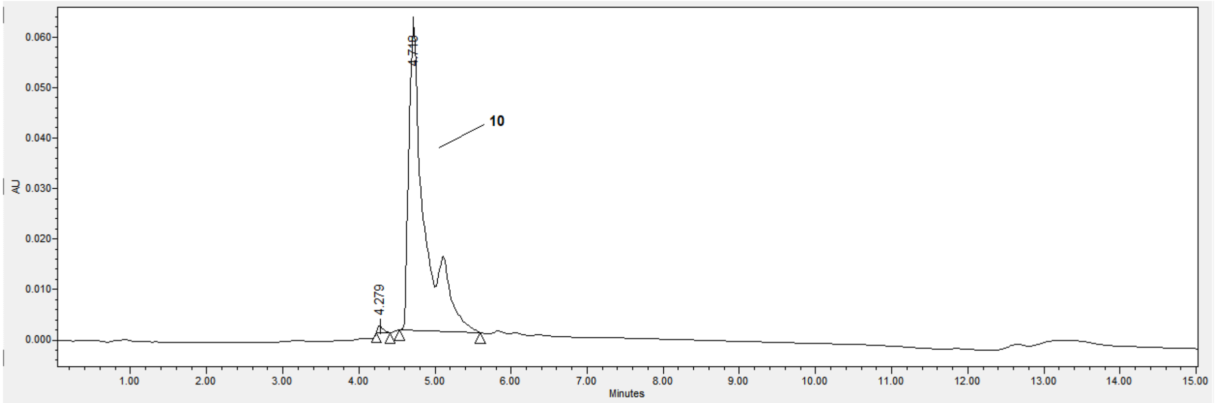

Figure S3 Analytical RP-HPLC chromatograms of compound **10.**

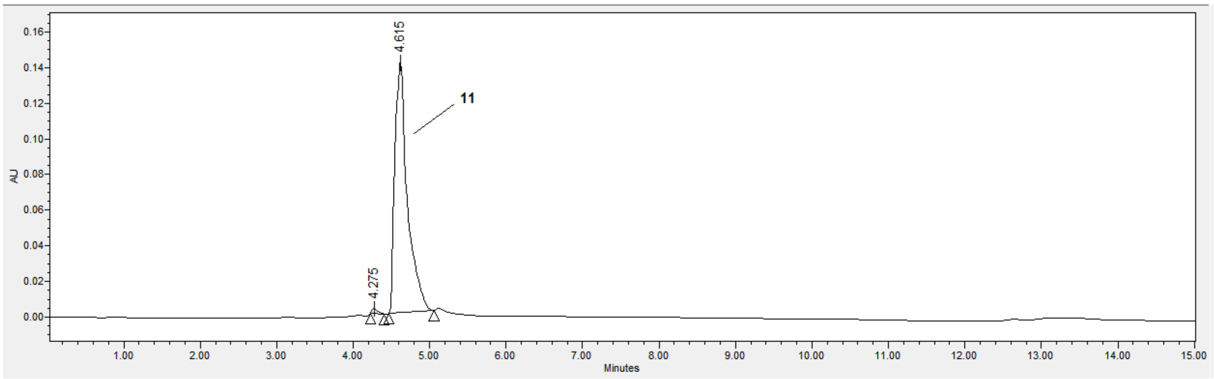

Figure S4 Analytical RP-HPLC chromatograms of compound **11.**

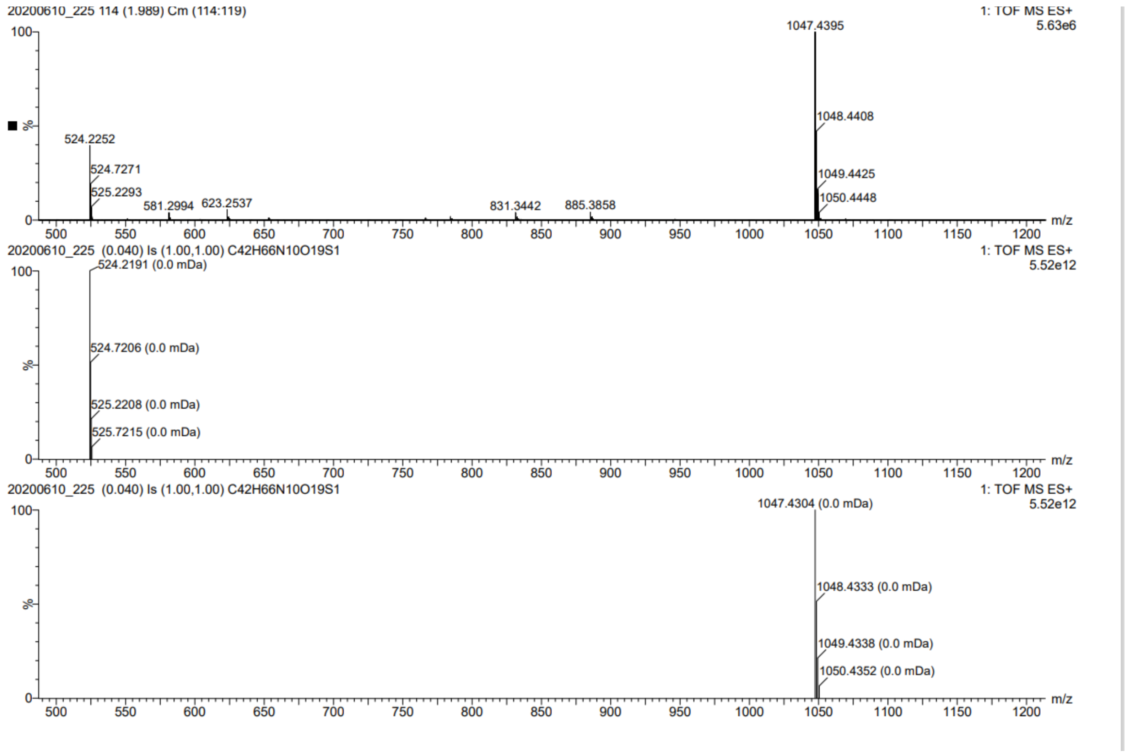

Figure S5 Mass spectrum of compound **3**.

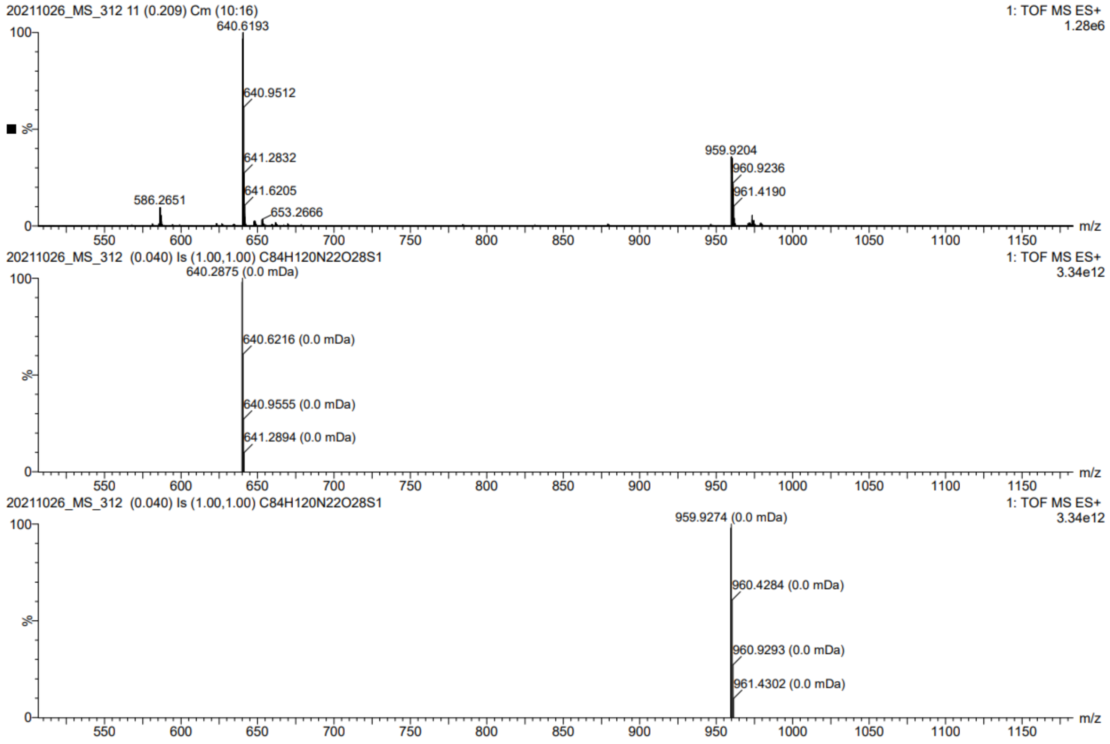

Figure S6 Mass spectrum of compound **9**.

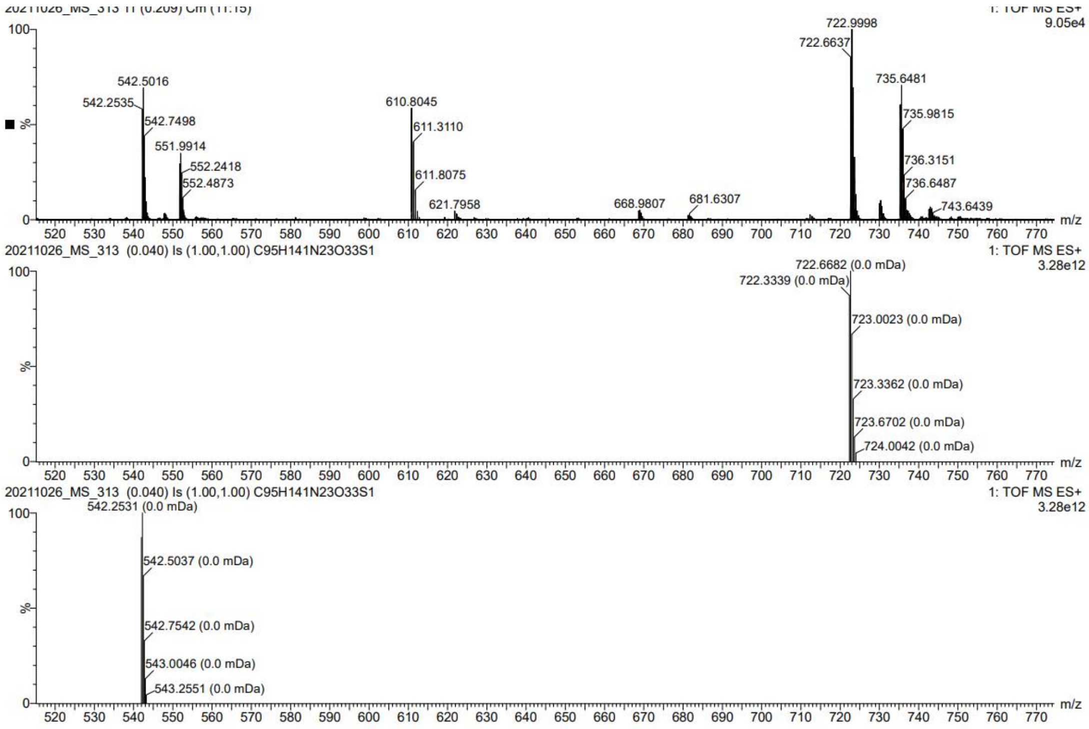

Figure S7 Mass spectrum of compound **10**.

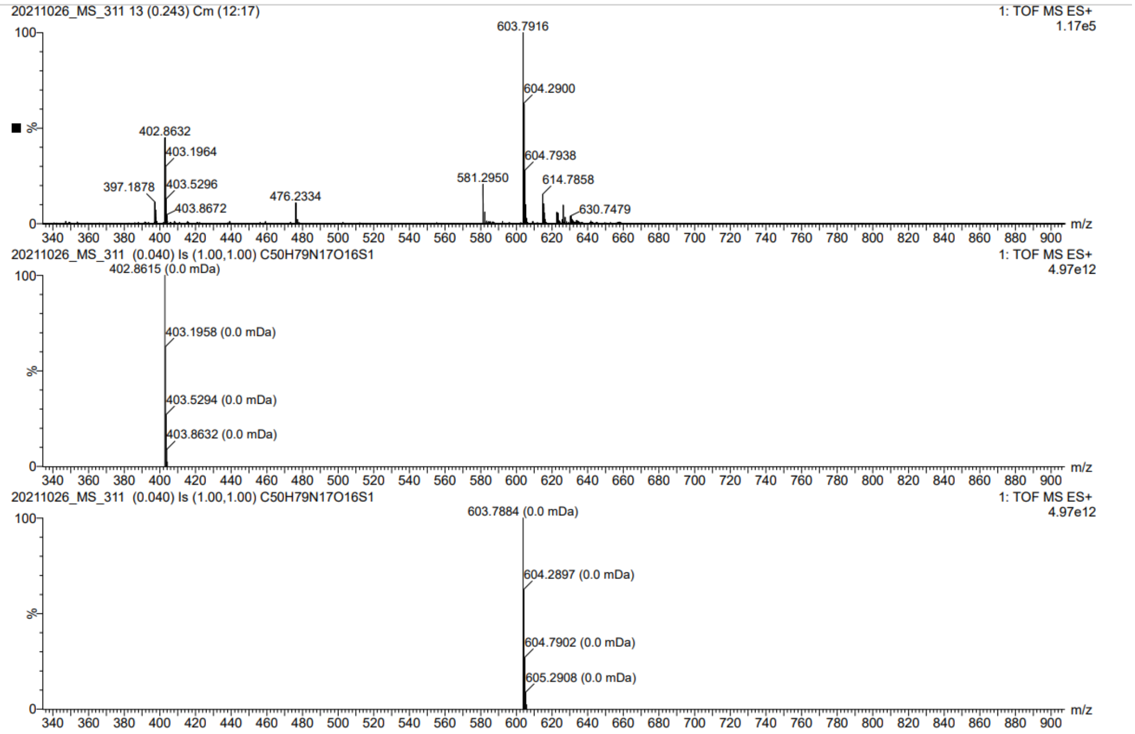

Figure S8 Mass spectrum of compound **11.**

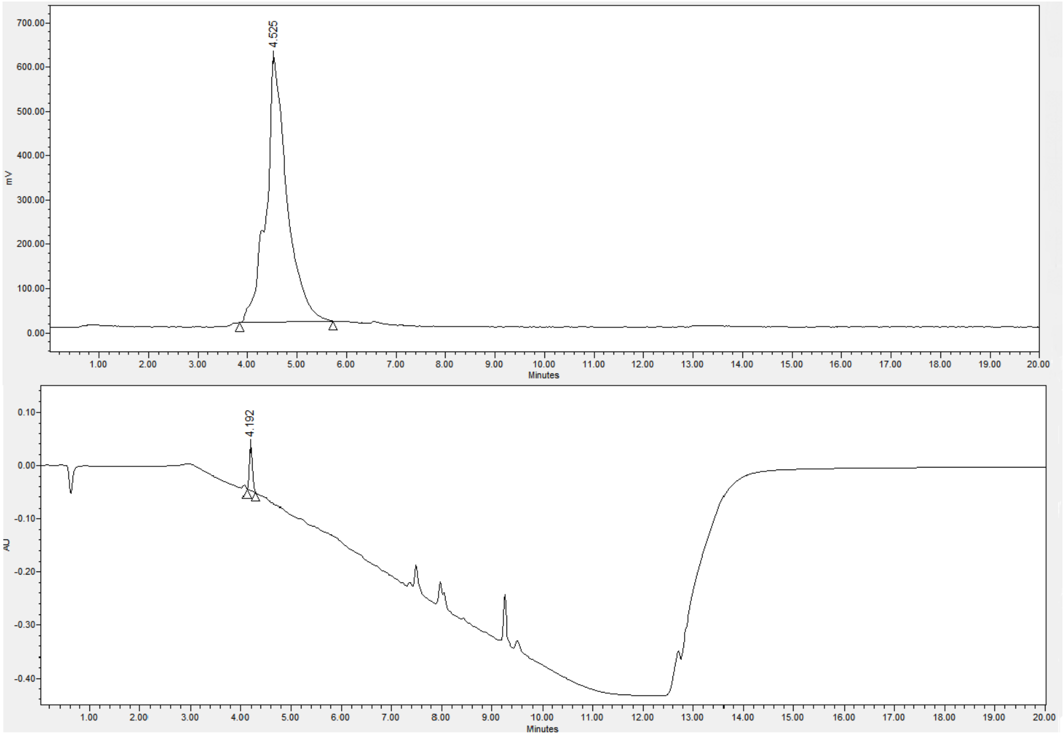

Figure S9 UV- and radio-HPLC chromatograms of [68Ga]-3.

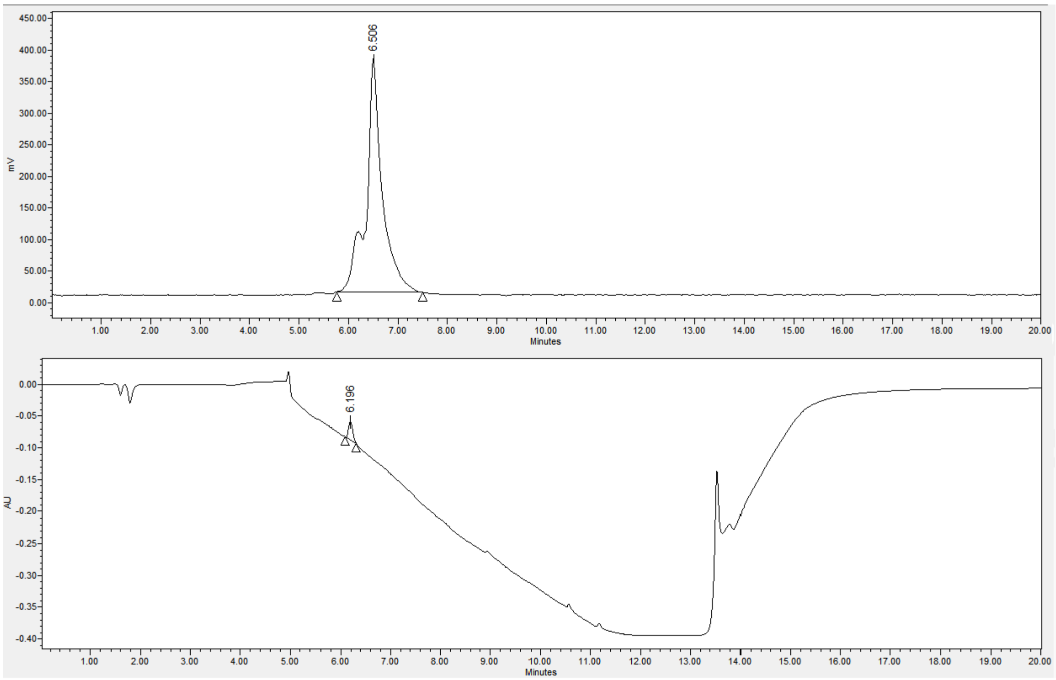

Figure S10 UV- and radio-HPLC chromatograms of [68Ga]-9.

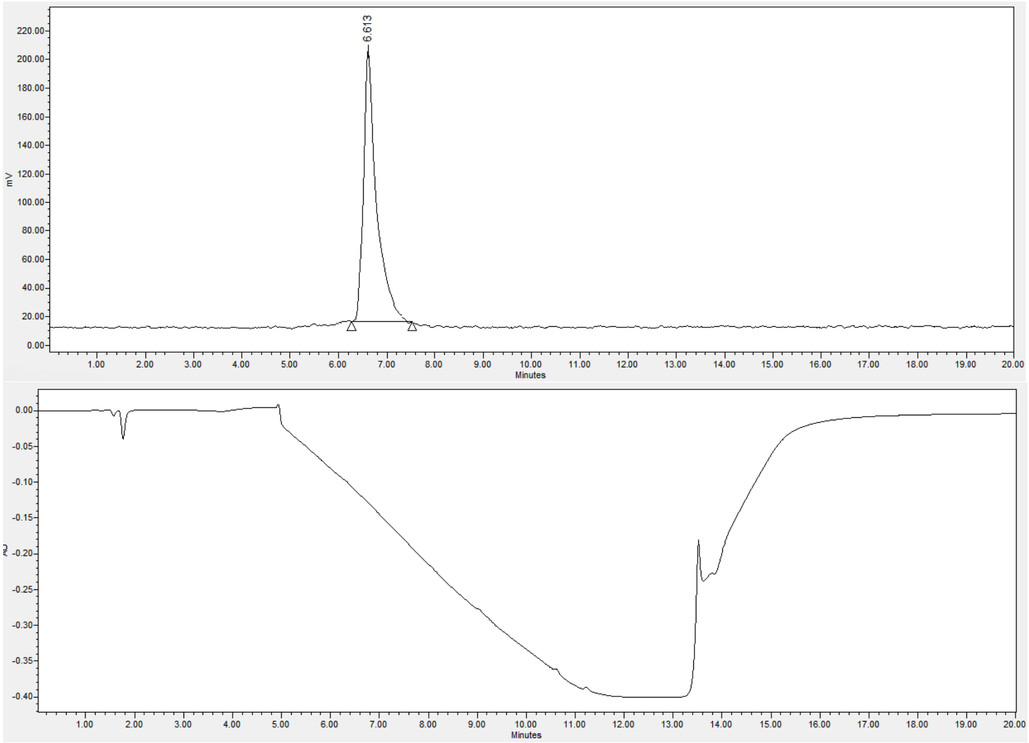

Figure S11 UV- and radio-HPLC chromatograms of [^{68}Ga]-10.

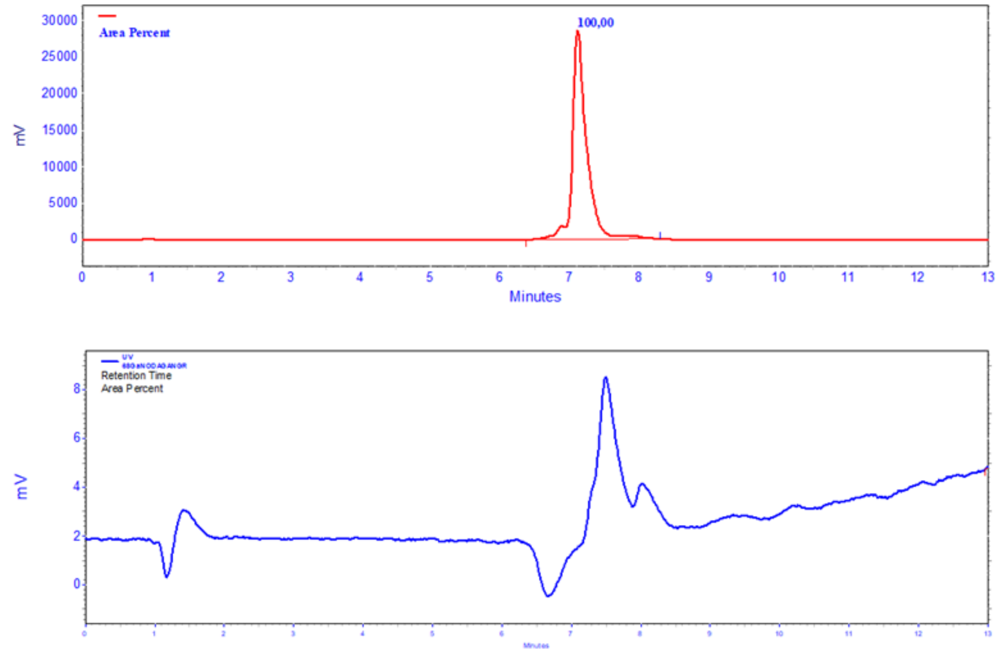

Figure S12 UV- and radio-HPLC chromatograms of [^{68}Ga]-**11.**

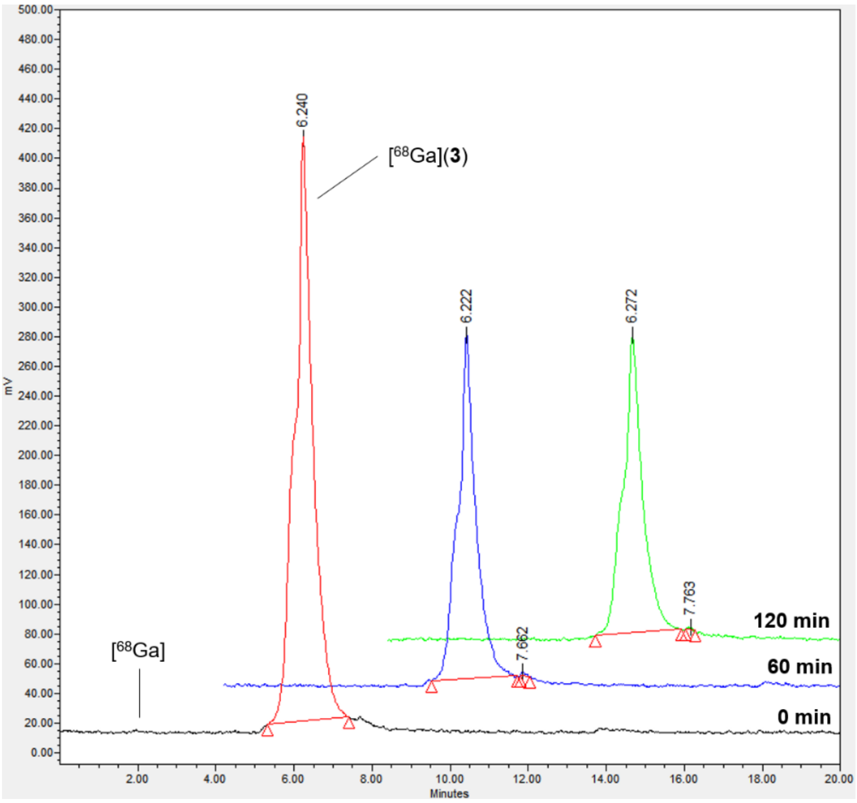

Figure S13 Radio-HPLC chromatograms of serum stability test of [68Ga]-3.

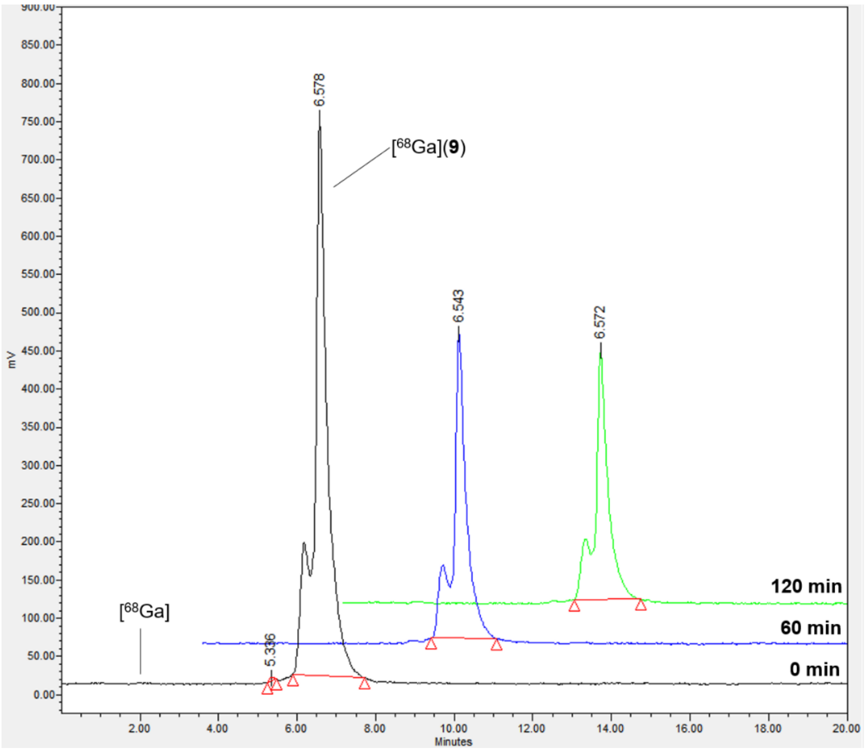

Figure S14 Radio-HPLC chromatograms of serum stability test of [68Ga]-9.

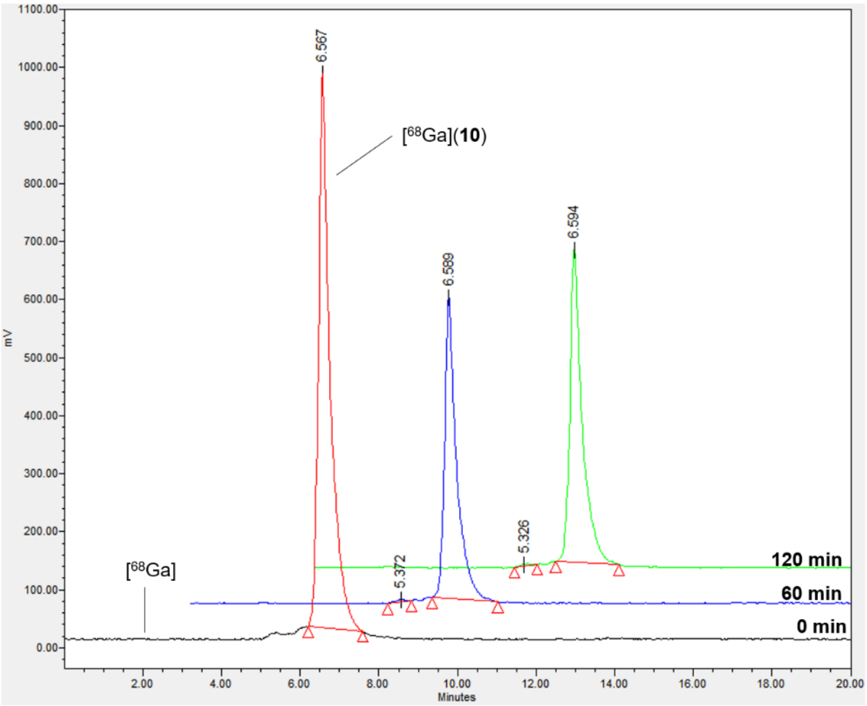

Figure S15 Radio-HPLC chromatograms of serum stability test of [^{68}Ga]-10.

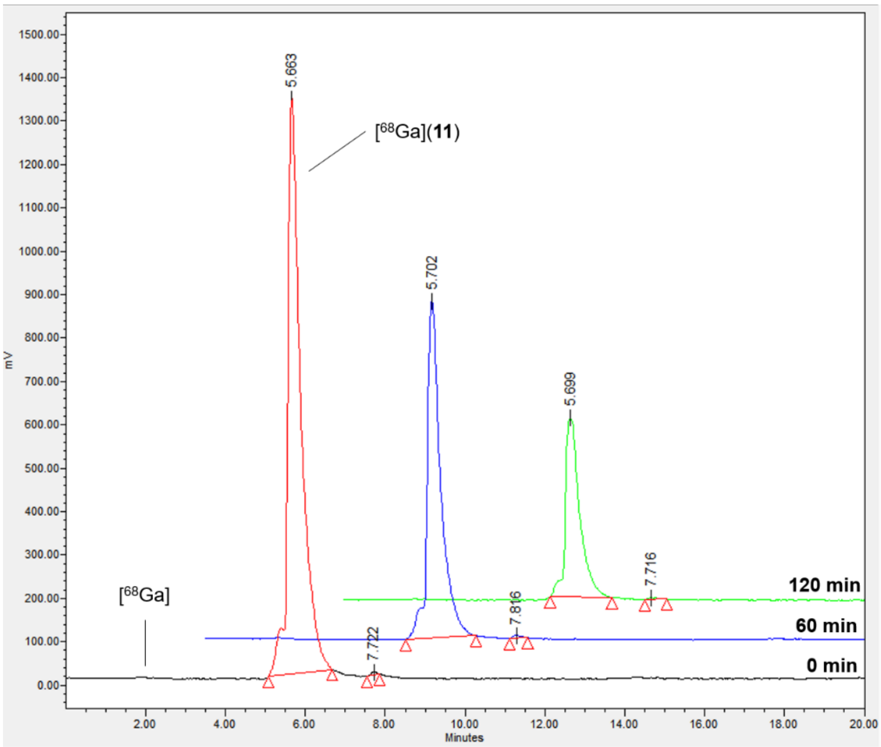

Figure S16 Radio-HPLC chromatograms of serum stability test of [^{68}Ga]-11.
